# Risk stratification of composite adverse outcomes after percutaneous nephrolithotomy based on preoperative inflammatory–nutritional status and stone complexity: a single-center retrospective study

**DOI:** 10.3389/fnut.2026.1867379

**Published:** 2026-07-22

**Authors:** Yu Qiu, Decai Ji, Guowang Du

**Affiliations:** 1 Urologic Oncology Center, Cangzhou Central Hospital, Cangzhou, China; 2 The No. 1 Department of Urology, Cangzhou People's Hospital, Cangzhou, China

**Keywords:** composite adverse outcomes, inflammatory–nutritional status, percutaneous nephrolithotomy, risk stratification, stone complexity

## Abstract

**Objective:**

To investigate the predictive value of preoperative inflammatory–nutritional status and stone complexity for composite adverse outcomes following percutaneous nephrolithotomy (PCNL), and to develop a clinical risk stratification model.

**Methods:**

Clinical data of 386 patients with renal calculi who underwent PCNL at the Cangzhou Central Hospital between January 2021 and December 2024 were retrospectively analyzed. Composite adverse outcomes were defined as the occurrence of any of the following: postoperative fever (temperature >38 °C), systemic inflammatory response syndrome (SIRS), requirement for blood transfusion, or complications of Clavien–Dindo grade ≥ II. Preoperative inflammatory–nutritional status was assessed using the neutrophil-to-lymphocyte ratio (NLR) and prognostic nutritional index (PNI), while stone complexity was quantified using the Guy’s grading system and the S. T. O. N. E. nephrolithometry score. Multivariate logistic regression analysis was performed to identify independent risk factors, and a risk stratification model was constructed based on the number of these factors.

**Results:**

Among the 386 patients, 121 (31.3%) experienced composite adverse outcomes postoperatively. Multivariate logistic regression analysis identified diabetes mellitus (OR = 2.41, 95% CI: 1.32–4.41, *p* = 0.004), preoperative urinary tract infection (OR = 3.27, 95% CI: 1.58–6.78, *p* = 0.001), NLR ≥ 3.5 (OR = 2.18, 95% CI: 1.35–3.52, *p* = 0.001), PNI < 45 (OR = 2.03, 95% CI: 1.22–3.38, *p* = 0.006), Guy’s grade ≥ III (OR = 2.67, 95% CI: 1.56–4.56, *p* < 0.001), and S. T. O. N. E. score ≥8 (OR = 3.14, 95% CI: 1.89–5.22, *p* < 0.001) as independent risk factors for composite adverse outcomes after PCNL. Receiver operating characteristic (ROC) curve analysis indicated that ≥3 risk factors was the optimal cutoff for predicting composite adverse outcomes, with a sensitivity of 78.5%, specificity of 75.2%, and an area under the curve (AUC) of 0.837 (95% CI: 0.791–0.883). Patients were stratified into low-risk (0–2 factors), intermediate-risk (3–4 factors), and high-risk (≥5 factors) groups, with corresponding incidence rates of composite adverse outcomes of 12.5, 34.6, and 68.4%, respectively (*p* < 0.001).

**Conclusion:**

Preoperative inflammatory–nutritional status (NLR, PNI) and stone complexity (Guy’s grading system, S. T. O. N. E. score) are independent predictors of composite adverse outcomes after PCNL. The risk stratification model based on the number of risk factors is simple and reliable, and can effectively identify high-risk patients, providing a basis for precise perioperative management.

## 1 Introduction

Urinary tract calculi are a common urological disease in China, with a prevalence of approximately 6.5% among adults, showing a rising trend year by year (1). Percutaneous nephrolithotomy (PCNL), as the gold standard for the treatment of complex renal stones, offers advantages such as a high stone-free rate and minimal invasiveness. However, the incidence of postoperative complications remains non-negligible. Previous studies have reported that the complication rate after PCNL ranges from 29.3 to 83.0%, mainly including fever, systemic inflammatory response syndrome (SIRS), bleeding, and urosepsis, which can be life-threatening in severe cases (2, 3). Therefore, accurate preoperative identification of high-risk patients and implementation of targeted interventions are of great importance for improving patient outcomes.

Previous studies have shown that adverse outcomes after PCNL are associated with multiple factors, including patient comorbidities (such as diabetes mellitus), preoperative urinary tract infection, stone burden, and surgical factors (4, 5). However, most existing studies have focused on single outcome measures (such as infection or bleeding), lacking a comprehensive evaluation of composite adverse outcomes. In recent years, the role of inflammatory–nutritional status in surgical prognosis has attracted increasing attention. The neutrophil-to-lymphocyte ratio (NLR) reflects the balance between systemic inflammatory response and immune status and has been demonstrated to be associated with postoperative infectious complications in various surgical settings (6). The prognostic nutritional index (PNI), which integrates serum albumin levels and peripheral lymphocyte count, reflects both nutritional reserves and immune status, and has recently shown promising predictive value in studies of urolithiasis surgery outcomes (7). Meanwhile, stone complexity scoring systems, such as the Guy’s grading system and the S. T. O. N. E. nephrolithometry score, can objectively quantify surgical difficulty and postoperative risk, and have been widely used in preoperative assessment for PCNL (8, 9).

However, there is still a lack of studies that combine host inflammatory–nutritional status with local stone characteristics to predict composite adverse outcomes after PCNL. Therefore, this study aims to investigate the predictive value of preoperative inflammatory–nutritional status indicators (NLR and PNI) and stone complexity (Guy’s grading system and S. T. O. N. E. score) for composite adverse outcomes following PCNL, and to construct a simple and practical risk stratification model, in order to provide a reference for clinical decision-making.

## 2 Materials and methods

### 2.1 Study population

A retrospective analysis was conducted on the clinical data of patients with renal calculi who underwent percutaneous nephrolithotomy (PCNL) in the Department of Urology at the Cangzhou Central Hospital from January 2021 to December 2024. The inclusion criteria were as follows: (1) age ≥18 years; (2) renal calculi or upper ureteral calculi confirmed by preoperative imaging; (3) first-time PCNL; and (4) complete clinical data. The exclusion criteria were: (1) severe cardiac, pulmonary, hepatic, or renal insufficiency; (2) coagulation disorders; (3) pregnancy or lactation; (4) simultaneous bilateral PCNL or concomitant surgeries; and (5) preexisting systemic infection or sepsis before surgery.

A total of 386 patients were included, comprising 235 males (60.9%) and 151 females (39.1%), aged 22–78 years, with a mean age of (51.4 ± 13.2) years. This study was approved by the Ethics Committee of Cangzhou Central Hospital (Approval No.: 2022–112-12). Given the retrospective nature of the study, informed consent was waived.

### 2.2 Surgical procedure

All procedures were performed by the same surgical team under general anesthesia. After adequate anesthesia, patients were placed in the lithotomy position. A cystoscope was inserted through the urethra, and a ureteral catheter was advanced into the ureter on the affected side with saline infusion. After catheter placement, the patient was repositioned to the prone position.

Following routine disinfection and draping, the target calyx was identified and punctured under ultrasound guidance. After successful puncture, a guidewire was inserted, and a percutaneous nephrostomy tract (F10–24) was established through serial dilation along the guidewire, followed by placement of a working sheath. Lithotripsy was performed using a rigid or flexible ureteroscope with a holmium laser. Larger stones were extracted using forceps, and continuous irrigation was maintained with a perfusion pump during the procedure.

After lithotripsy, the renal pelvis, calyces, and upper ureter were carefully inspected to confirm the absence of residual stones. A double-J ureteral stent (F4.7/6.0) was placed, and a nephrostomy tube was inserted and secured. The equipment used included a holmium laser lithotripsy system (200 μm fiber), rigid ureteroscope and URF-P6 flexible ureteroscope (F4.9/7.95, Olympus, Japan), COOK hydrophilic guidewire (Cook Medical, USA), and double-J ureteral stents (Cook Medical, USA).

### 2.3 Data collection

Clinical data were collected, including baseline characteristics, laboratory parameters, stone-related variables, perioperative parameters, and postoperative outcomes. Baseline characteristics included age, sex, body mass index (BMI), smoking history, history of diabetes mellitus, and hypertension.

Laboratory parameters included preoperative complete blood count, C-reactive protein (CRP), and albumin (Alb). The following indices were calculated: neutrophil-to-lymphocyte ratio (NLR) = neutrophil count / lymphocyte count; C-reactive protein-to-albumin ratio (CAR) = CRP / Alb; prognostic nutritional index (PNI) = Alb (g/L) + 5 × lymphocyte count (×10^9^/L).

Stone-related variables included stone location, maximum diameter, staghorn calculi, multiple stones, Guy’s grading system, and S. T. O. N. E. nephrolithometry score. Surgical variables included operative time, number of access tracts, and stone-free rate. Postoperative complications were graded according to the Clavien–Dindo classification system (10).

### 2.4 Definition of composite adverse outcomes

Preoperative urinary tract infection was defined as a positive midstream urine culture or ≥10 leukocytes per high-power field on urinalysis. Diabetes mellitus was defined as a prior diagnosis or current use of antidiabetic medications. Hypertension was defined as systolic blood pressure ≥140 mmHg and/or diastolic blood pressure ≥90 mmHg, or a prior diagnosis with ongoing antihypertensive treatment.

Composite adverse outcomes were defined as the occurrence of any of the following events after surgery: fever (body temperature >38.0 °C within 48 h postoperatively); systemic inflammatory response syndrome (SIRS), defined by the presence of at least two of the following criteria—body temperature >38 °C or <36 °C, heart rate >90 beats/min, respiratory rate >20 breaths/min or PaCO₂ < 32 mmHg, white blood cell count >12 × 10^9^/L or <4 × 10^9^/L (11); requirement for blood transfusion; or complications of Clavien–Dindo grade ≥ II (10).

Stone-free status was defined as no residual stones or residual fragments <4 mm on postoperative imaging (KUB or CT).

### 2.5 Statistical analysis

Statistical analysis was performed using SPSS version 26.0. Continuous variables were first tested for normality using the Shapiro–Wilk test. Normally distributed data were expressed as mean ± standard deviation (x̄ ± s), and comparisons between groups were performed using the independent samples t-test. Non-normally distributed data were expressed as median (Q₁, Q₃), and comparisons were conducted using the Mann–Whitney U test.

Categorical variables were expressed as frequencies (%), and comparisons were performed using the chi-square test or Fisher’s exact test. The optimal cutoff values for NLR and PNI were determined using receiver operating characteristic (ROC) curve analysis based on the maximum Youden index.

Multicollinearity among variables was assessed using the variance inflation factor (VIF), with VIF < 5 indicating no significant collinearity. Multivariate logistic regression analysis was used to identify independent risk factors for composite adverse outcomes, with *p* < 0.05 considered statistically significant. A risk stratification model was constructed based on the regression results, and its predictive performance was evaluated using ROC curve analysis.

## 3 Results

### 3.1 Baseline characteristics and incidence of adverse outcomes

Among the 386 patients, 121 (31.3%) developed composite adverse outcomes after surgery. Specifically, 78 patients (20.2%) experienced fever, 45 (11.7%) developed systemic inflammatory response syndrome (SIRS), 22 (5.7%) required blood transfusion, and 35 (9.1%) had complications of Clavien–Dindo grade ≥ II. Some patients experienced more than one adverse event.

Patients were divided into the adverse outcome group (*n* = 121) and the control group (*n* = 265) according to the occurrence of composite adverse outcomes. The comparison of baseline characteristics between the two groups is shown in Table 1. The results demonstrated that the proportions of diabetes mellitus (32.2% vs. 18.1%, *p* = 0.002) and preoperative urinary tract infection (38.8% vs. 20.4%, *p* < 0.001) were significantly higher in the adverse outcome group than in the control group.

**Table 1:** Comparison of baseline characteristics between the two groups.

Variable	Adverse outcome group (*n* = 121)	Control group (*n* = 265)	Statistic (t/χ^2^/Z)	*p* value
General characteristics				
Age (years, x̄ ± s)	53.2 ± 13.8	50.6 ± 12.9	1.824	0.069
Male [*n* (%)]	76 (62.8)	159 (60.0)	0.275	0.6
BMI (kg/m^2^, x̄ ± s)	24.8 ± 3.1	24.3 ± 2.9	1.553	0.121
Smoking history [*n* (%)]	38 (31.4)	71 (26.8)	0.869	0.351
Diabetes mellitus [*n* (%)]	39 (32.2)	48 (18.1)	9.53	0.002
Hypertension [*n* (%)]	44 (36.4)	80 (30.2)	1.467	0.226
Preoperative laboratory parameters				
Preoperative UTI [*n* (%)]	47 (38.8)	54 (20.4)	14.885	<0.001
Hemoglobin (g/L, x̄ ± s)	124.6 ± 15.3	128.9 ± 14.1	−2.705	0.007
CRP [mg/L, M (Q₁, Q₃)]	8.2 (3.1, 18.5)	4.1 (1.8, 9.3)	−4.987	<0.001
Albumin (g/L, x̄ ± s)	38.2 ± 4.5	40.1 ± 3.9	−4.221	<0.001
Lymphocyte count (×10^9^/L, x̄ ± s)	1.52 ± 0.48	1.78 ± 0.52	−4.587	<0.001
NLR (x̄ ± s)	3.8 ± 1.6	2.9 ± 1.2	6.149	<0.001
CAR (x̄ ± s)	0.35 ± 0.22	0.24 ± 0.18	5.272	<0.001
PNI (x̄ ± s)	44.6 ± 6.2	48.9 ± 5.7	−6.701	<0.001
Stone characteristics				
Maximum stone diameter (cm, x̄ ± s)	3.4 ± 1.1	2.6 ± 0.9	7.479	<0.001
Staghorn calculi [*n* (%)]	45 (37.2)	62 (23.4)	7.999	0.005
Multiple stones [*n* (%)]	52 (43.0)	88 (33.2)	3.476	0.062
Guy’s grade ≥ III [*n* (%)]	82 (67.8)	109 (41.1)	23.556	<0.001
S. T. O. N. E. score (x̄ ± s)	7.9 ± 1.4	6.8 ± 1.3	7.676	<0.001
Surgical variables				
Operative time (min, x̄ ± s)	105.6 ± 28.4	89.3 ± 24.7	5.841	<0.001
Multiple tracts [*n* (%)]	18 (14.9)	21 (7.9)	4.306	0.038
Stone-free rate [*n* (%)]	91 (75.2)	220 (83.0)	3.279	0.07

NLR = neutrophil-to-lymphocyte ratio; CAR = C-reactive protein-to-albumin ratio; PNI = prognostic nutritional index; CRP = C-reactive protein.

Regarding inflammatory–nutritional indicators, the adverse outcome group had significantly higher NLR (3.8 ± 1.6 vs. 2.9 ± 1.2, *p* < 0.001) and CAR (0.35 ± 0.22 vs. 0.24 ± 0.18, *p* < 0.001), and significantly lower PNI (44.6 ± 6.2 vs. 48.9 ± 5.7, *p* < 0.001).

In terms of stone characteristics, the adverse outcome group had significantly higher rates of Guy’s grade ≥ III (67.8% vs. 41.1%, *p* < 0.001), higher S. T. O. N. E. scores (7.9 ± 1.4 vs. 6.8 ± 1.3, *p* < 0.001), and larger maximum stone diameter (3.4 ± 1.1 cm vs. 2.6 ± 0.9 cm, *p* < 0.001) compared with the control group.

### 3.2 Multivariate logistic regression analysis of composite adverse outcomes

Variables with statistical significance in univariate analysis were included in the multivariate logistic regression model. Collinearity diagnostics showed that all variables had variance inflation factors (VIF) < 2.5, indicating no significant multicollinearity.

The results demonstrated that diabetes mellitus (OR = 2.41, 95% CI: 1.32–4.41, *p* = 0.004), preoperative urinary tract infection (OR = 3.27, 95% CI: 1.58–6.78, *p* = 0.001), NLR ≥ 3.5 (OR = 2.18, 95% CI: 1.35–3.52, *p* = 0.001), PNI < 45 (OR = 2.03, 95% CI: 1.22–3.38, *p* = 0.006), Guy’s grade ≥ III (OR = 2.67, 95% CI: 1.56–4.56, *p* < 0.001), and S. T. O. N. E. score ≥8 (OR = 3.14, 95% CI: 1.89–5.22, *p* < 0.001) were independent risk factors for composite adverse outcomes after PCNL (Table 2).

**Table 2:** Multivariate logistic regression analysis of composite adverse outcomes after PCNL.

Variable	β	SE	Wald χ^2^	OR (95% CI)	*p* value
Diabetes mellitus (Yes vs. No)	0.879	0.307	8.192	2.41 (1.32–4.41)	0.004
Preoperative UTI (Yes vs. No)	1.185	0.371	10.202	3.27 (1.58–6.78)	0.001
NLR ≥ 3.5 (Yes vs. No)	0.779	0.246	10.022	2.18 (1.35–3.52)	0.001
PNI < 45 (Yes vs. No)	0.708	0.259	7.47	2.03 (1.22–3.38)	0.006
Guy’s grade ≥ III (Yes vs. No)	0.982	0.273	12.931	2.67 (1.56–4.56)	<0.001
S. T. O. N. E. score ≥8 (Yes vs. No)	1.145	0.26	19.365	3.14 (1.89–5.22)	<0.001

OR = odds ratio; CI = confidence interval.

### 3.3 Construction and validation of the risk stratification model

A risk stratification model was developed based on the six independent risk factors identified in the multivariate analysis (diabetes mellitus, preoperative urinary tract infection, NLR ≥ 3.5, PNI < 45, Guy’s grade ≥ III, and S. T. O. N. E. score ≥8).

**Figure 1:**
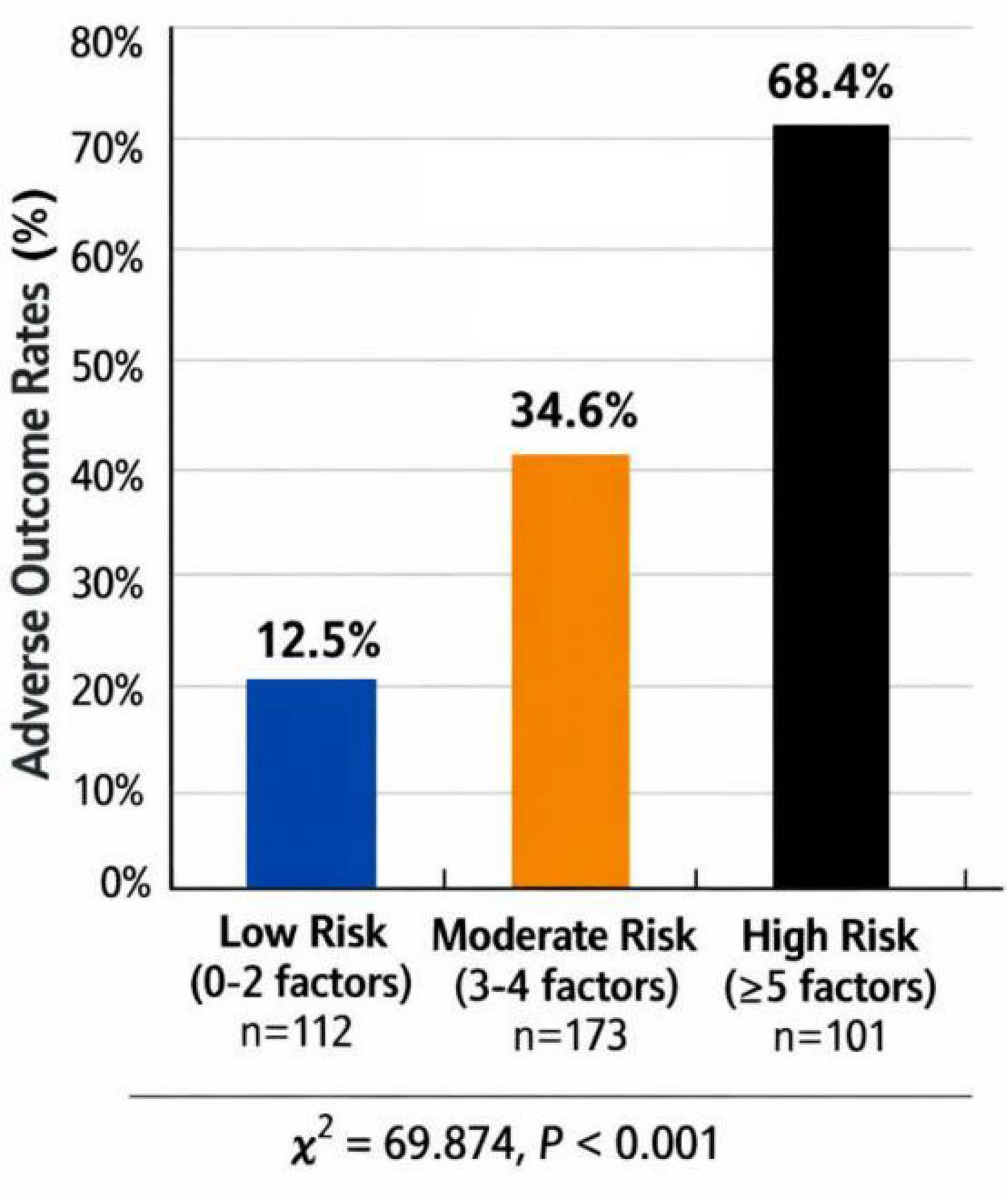
Comparison of the incidence of composite adverse outcomes among different risk groups. Patients were divided into low-risk (0–2 risk factors, *n* = 112), intermediate-risk (3–4 risk factors, *n* = 173), and high-risk (≥5 risk factors, *n* = 101) groups. The incidence rates of composite adverse outcomes were 12.5, 34.6, and 68.4%, respectively, with statistically significant differences among groups (χ^2^ = 69.874, *p* < 0.001).

Patients were stratified into three groups according to the number of risk factors: low-risk group (0–2 factors), intermediate-risk group (3–4 factors), and high-risk group (≥5 factors). The incidence of composite adverse outcomes was 12.5% (14/112) in the low-risk group, 34.6% (60/173) in the intermediate-risk group, and 68.4% (69/101) in the high-risk group. The differences among the groups were statistically significant (χ^2^ = 69.874, *p* < 0.001; Figure 1).

**Figure 2:**
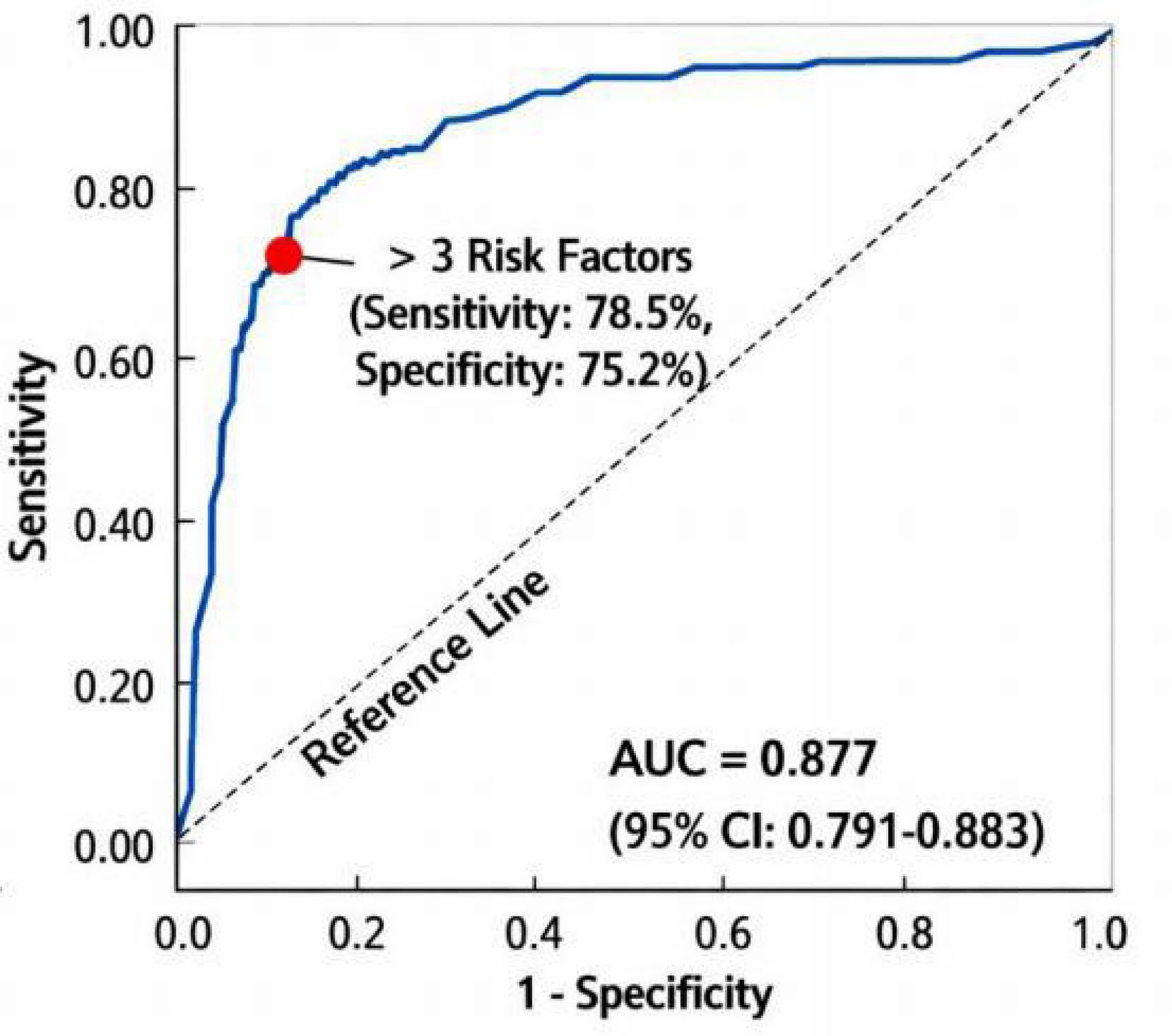
ROC curve of the risk stratification model for predicting composite adverse outcomes. The AUC for predicting composite adverse outcomes based on the number of risk factors was 0.837 (95% CI: 0.791–0.883). The optimal cutoff value was ≥3 risk factors, with a sensitivity of 78.5% and specificity of 75.2%.

Receiver operating characteristic (ROC) curve analysis showed that ≥3 risk factors was the optimal cutoff value for predicting composite adverse outcomes, with a sensitivity of 78.5%, specificity of 75.2%, and an area under the curve (AUC) of 0.837 (95% CI, 0.791–0.883, Figure 2), indicating good predictive performance of the model.

## 4 Discussion

In this study, a retrospective analysis of 386 patients undergoing PCNL was conducted to systematically evaluate the predictive value of preoperative inflammatory–nutritional status and stone complexity for postoperative composite adverse outcomes, and to develop a risk stratification model based on the number of risk factors. To our knowledge, this is the first study to integrate systemic inflammatory–nutritional indicators (NLR and PNI) with anatomical stone complexity scores (Guy’s grading system and S. T. O. N. E. score) for risk stratification of composite adverse outcomes after PCNL. Compared with previous studies focusing on a single outcome or a single dimension, this study incorporated both host-related and stone-related factors, providing a more comprehensive assessment of postoperative risk. The main findings are as follows: (1) the incidence of composite adverse outcomes after PCNL was 31.3%, consistent with previously reported complication rates; (2) diabetes mellitus, preoperative urinary tract infection, NLR ≥ 3.5, PNI < 45, Guy’s grade ≥ III, and S. T. O. N. E. score ≥8 were independent risk factors; and (3) the risk stratification model based on these factors effectively distinguished patients at different risk levels, with an incidence of adverse outcomes as high as 68.4% in the high-risk group.

Diabetes mellitus is a well-recognized risk factor for postoperative complications after PCNL. In the present study, diabetes increased the risk of composite adverse outcomes by 2.41-fold, which is consistent with previous findings (11). Patients with diabetes often have impaired immune function, microvascular disease, and delayed wound healing, making them more susceptible to complications such as infection and bleeding. Preoperative urinary tract infection was also identified as an important risk factor, with an OR of 3.27, indicating that even after preoperative anti-infective treatment, the risk of infection-related complications remains significantly elevated. Previous studies have suggested that renal pelvic urine culture and stone culture are better predictors of postoperative SIRS after PCNL (12), indicating that obtaining renal pelvic urine and stone specimens intraoperatively for culture may help guide postoperative antibiotic therapy in patients with preoperative infection.

Inflammatory–nutritional status is a key focus of this study. NLR, as an important marker reflecting systemic inflammatory response, has been shown to be associated with postoperative complications in various surgical settings. In this study, NLR ≥ 3.5 increased the risk of composite adverse outcomes by 2.18-fold, consistent with the findings of Kriplani et al. (13). An elevated NLR indicates a relative increase in neutrophils and a decrease in lymphocytes; the former mediates inflammatory responses, while the latter reflects immune status. This imbalance suggests reduced tolerance to surgical stress.

PNI integrates serum albumin levels and peripheral lymphocyte count, reflecting both nutritional reserve and immune status. In this study, patients with PNI < 45 had a 2.03-fold increased risk of adverse outcomes, consistent with the findings of Noviardi et al. (14). Patients with low PNI often exhibit malnutrition and immunosuppression, which significantly increase the risk of postoperative infection and impaired wound healing. Notably, the Mayo Adhesive Probability (MAP) score, an imaging indicator used to assess perirenal fat adhesion, has recently been shown to be associated with postoperative infectious complications (15), suggesting that perirenal fat inflammatory status may also influence surgical outcomes and warrants further investigation.

Stone complexity is a key factor influencing surgical difficulty and prognosis in PCNL. In this study, Guy’s grade ≥ III and S. T. O. N. E. score ≥8 increased the risk of composite adverse outcomes by 2.67-fold and 3.14-fold, respectively. The Guy’s grading system is based on stone location and renal collecting system anatomy (16), whereas the S. T. O. N. E. score comprehensively evaluates stone size, tract length, degree of obstruction, stone density, and the number of involved calyces (17). Highly complex stones often require longer operative times, multiple access tracts, and higher irrigation volumes, thereby increasing the risk of infection and bleeding. However, some studies have reported no association between the S. T. O. N. E. score and postoperative complications (18), which may be related to differences in sample size and outcome definitions, indicating the need for further validation.

Interestingly, some studies have found that the absence of staghorn calculi and the presence of no or mild hydronephrosis may paradoxically increase the risk of severe postoperative bleeding (19). This may be explained by the fact that staghorn calculi are often associated with renal parenchymal atrophy and reduced vascularity, whereas normal renal tissue has a richer blood supply, leading to a higher risk of bleeding after puncture. This counterintuitive finding suggests that clinicians should consider multiple factors when assessing bleeding risk rather than assuming that higher stone complexity always corresponds to higher risk.

The risk stratification model developed in this study integrates inflammatory–nutritional status and stone complexity, enabling accurate prediction of composite adverse outcomes after PCNL. Unlike models such as the CROES nomogram, which primarily predict stone-free rates, this study focuses on postoperative complications—an important clinical outcome—and includes only preoperatively available variables, making it more suitable for preoperative risk assessment and doctor–patient communication (20). The incidence of adverse outcomes in the high-risk group (68.4%) was significantly higher than that in the low-risk group (12.5%), highlighting the clinical importance of identifying high-risk patients preoperatively and implementing targeted interventions. For high-risk patients, the following measures may be considered: (1) strict perioperative glycemic control; (2) adequate anti-infective treatment, with prolonged preoperative antibiotic use if necessary; (3) nutritional support to improve preoperative nutritional status; (4) careful selection of surgical timing, with staged procedures if needed; and (5) enhanced postoperative monitoring for early detection and management of complications.

This study has several limitations. First, as a single-center retrospective study, selection bias is inevitable, and the findings require validation in multicenter prospective studies. Second, the definition of composite adverse outcomes includes multiple events, which reflects overall risk but may obscure differences in risk factors for individual outcomes; future studies could perform subgroup analyses for specific endpoints. In addition, intraoperative factors (such as irrigation pressure and puncture experience) and postoperative antibiotic use were not included, although these may also influence prognosis. Finally, intraoperative dynamic parameters (such as irrigation pressure, tract location, and lithotripsy energy) were not evaluated, although they may independently affect the risk of postoperative infection and bleeding. Future studies incorporating real-time intraoperative data may enable the development of dynamic prediction models with improved accuracy.

## 5 Conclusion

Preoperative inflammatory–nutritional status (NLR and PNI) and stone complexity (Guy’s grading system and S. T. O. N. E. score) are independent predictors of composite adverse outcomes after PCNL. The risk stratification model based on the number of risk factors is simple and reliable, and can effectively identify high-risk patients, providing a basis for precise perioperative management. For high-risk patients with multiple risk factors, enhanced comprehensive perioperative management should be implemented to reduce the risk of postoperative complications.

## Data Availability

The original contributions presented in the study are included in the article/supplementary material, further inquiries can be directed to the corresponding author.

## References

[1] Wang Z Zhang Y Zhang J Deng Q Liang H. Recent advances on the mechanisms of kidney stone formation (review). Int J Mol Med. (2021) 48:149. doi: 10.3892/ijmm.2021.4982, 34132361 PMC8208620

[2] Tailly T Tsaturyan A Emiliani E Somani B Pietropaolo A Ozsoy M . Worldwide practice patterns of percutaneous nephrolithotomy. World J Urol. (2022) 40:2091–8. doi: 10.1007/s00345-022-04067-3, 35776174

[3] Choong S de la Rosette J Denstedt J Zeng G Sarica K Mazzon G . Classification and Standardized Reporting of Percutaneous Nephrolithotomy (PCNL): International Alliance of Urolithiasis (IAU) consensus Statements. Minerva Urol Nephrol. (2022) 74:110–8. doi: 10.23736/S2724-6051.20.04107-7 33439573

[4] Zhou G Zhou Y Chen R Wang D Zhou S Zhong J . The influencing factors of infectious complications after percutaneous nephrolithotomy: a systematic review and meta-analysis. Urolithiasis. (2022) 51:17. doi: 10.1007/s00240-022-01376-5, 36515726 PMC9750925

[5] Gelmis M Bulut B Kose MG Gonultas S Ayten A Arslan B. Evaluating postoperative complications in standard percutaneous nephrolithotomy for renal stones larger than 2 cm: a retrospective study utilizing the E-PASS scoring system. Urolithiasis. (2025) 53:20. doi: 10.1007/s00240-024-01689-7, 39777505

[6] Wei Q Liu AM Sun ZY Zhang S Hao ZY. The predictive value of systemic inflammatory biomarkers in predicting postoperative systemic inflammatory response syndrome after percutaneous nephrolithotomy. Int J Gen Med. (2024) 17:6513–21. doi: 10.2147/IJGM.S497322, 39749258 PMC11693952

[7] Qi S Huang S Qian RJJoE. Predictive value of combined serum nuclear factor erythroid 2-related factor 2 and prognostic nutritional index for sepsis after percutaneous nephrolithotomy. Sage Journals (2025) 39:222–30. doi: 10.1089/end.2024.0519, 39964774

[8] Mushtaq A Wani S Shaheen FA Hamid A Khawaja AR Malik SA . Evaluation of the GUYS, STONE, and S-ReSC scores in predicting the outcomes of mini-percutaneous nephrolithotomy: a prospective study. Cureus (2025) 17:e82723. doi: 10.7759/cureus.82723, 40400872 PMC12094496

[9] Farooq K Hameed N Zaib Z Hameed MB Ausaf H Shakil F . Comparison of STONE score, guy’s stone score, CROES nomogram, and Seoul national university renal stone complexity score in prognosticating outcomes of multiple-tract mini-percutaneous nephrolithotomy: a retrospective study. Cureus (2024) 16:e54790. doi: 10.7759/cureus.54790, 38529424 PMC10961480

[10] Tzelves L Geraghty R Mourmouris P Chatzikrachtis N Karavitakis M Somani B . Shockwave lithotripsy complications according to modified Clavien-Dindo grading system. A systematic review and meta-regression analysis in a sample of 115 randomized controlled trials. Eur Urol Focus. (2022) 8:1452–60. doi: 10.1016/j.euf.2021.11.002, 34848163

[11] Yarimoglu S Bozkurt IH Aydogdu O Yonguc T Gunlusoy B Degirmenci T. External validation and comparisons of the scoring systems for predicting percutaneous nephrolithotomy outcomes: a single center experience with 506 cases. J Laparoendosc Adv Surg Tech A. (2017) 27:1284–9. doi: 10.1089/lap.2017.0355, 28873326

[12] Li Y Xie L Liu CJH. Prediction of systemic inflammatory response syndrome and urosepsis after percutaneous nephrolithotomy by urine culture, stone culture, and renal pelvis urine culture: systematic review and meta-analysis. Heliyon. (2024) 10:e33155. doi: 10.1016/j.heliyon.2024.e33155, 39040347 PMC11260937

[13] Kriplani A Pandit S Chawla A de la Rosette JJMCH Laguna P Jayadeva Reddy S . Neutrophil–lymphocyte ratio (NLR), platelet–lymphocyte ratio (PLR) and lymphocyte–monocyte ratio (LMR) in predicting systemic inflammatory response syndrome (SIRS) and sepsis after percutaneous nephrolithotomy (PNL). Urolithiasis. (2022) 50:341–8. doi: 10.1007/s00240-022-01319-0, 35246692 PMC9110452

[14] Noviardi DEPP Zuhirman Jaya I Afdal Pitoyo J Yashar MA . Preoperative inflammatory biomarkers analysis in prognosis of systemic inflammatory response syndrome following percutaneous nephrolithotomy: a systematic review and meta-analysis. Arab J Urol. (2023) 21:108–17. doi: 10.1080/2090598X.2022.2138891, 37426769 PMC10325112

[15] Xu R Wang JJ Zhao WH Xiong J Lu ZW Mo LC. Predicting postoperative fever in culture-negative patients undergoing mini-PCNL using MAP score-augmented machine learning: a retrospective cohort study. World J Urol. (2025) 43:575. doi: 10.1007/s00345-025-05952-3, 40996565 PMC12464075

[16] Lopez Silva M Sanguinetti H Padial Tagliapietra L Aguilar J Bernardo N . Is guy’s stone score useful for predicting outcomes in percutaneous nephrolithotomy? Actas Urológicas Españolas (English Edition) (2022) 46:92–7. doi: 10.1016/j.acuroe.2021.01.009, 35125338

[17] Ayranci A Erbin A Caglar U Meric A Gunay NF Sarilar O. Comparison of CROES, guy’s, STONE, and S-ReSC nephrolithometric scoring systems in predicting success and complications in patients undergoing supine mini percutaneous nephrolithotomy. Urolithiasis. (2024) 52:147. doi: 10.1007/s00240-024-01642-8, 39402269

[18] Choudhary A Jayadeva-Reddy S Shetty S Sourabh-Reddy B Singh A Irappa-Wali M. Evaluation of the STONE nephrolithometry score in predicting surgical outcomes of percutaneous nephrolithotomy: results of a prospective study at a university hospital. Revista mexicana de urología (2023) 83:1–13. doi: 10.48193/revistamexicanadeurologa.v83i3.922,

[19] Sun H Chen Z. Integration of minimally invasive techniques and interventional therapy: application of percutaneous nephrolithotomy in patients with upper urinary tract stones and an analysis of risk factors for postoperative bleeding. Front. Med (2025) 12:1556224. doi: 10.3389/fmed.2025.1556224, 40385576 PMC12081443

[20] Mazzon G Choong S Celia A. Stone-scoring systems for predicting complications in percutaneous nephrolithotomy: a systematic review of the literature. Asian Journal of Urology (2023) 10:226–38. doi: 10.1016/j.ajur.2023.01.005, 37538152 PMC10394284

